# The Nicotinic Acetylcholine Receptor Dα7 Is Required for an Escape Behavior in*Drosophila*

**DOI:** 10.1371/journal.pbio.0040063

**Published:** 2006-02-28

**Authors:** Amir Fayyazuddin, Mahira A Zaheer, P. Robin Hiesinger, Hugo J Bellen

**Affiliations:** **1**Howard Hughes Medical Institute, Baylor College of Medicine, Houston, Texas, United States of America; **2**Department of Molecular and Human Genetics, Baylor College of Medicine, Houston, Texas, United States of America; **3**Department of Neuroscience, Baylor College of Medicine, Houston, Texas, United States of America; **4**Program in Developmental Biology, Baylor College of Medicine, Houston, Texas, United States of America; Austrian Academy of SciencesAustria

## Abstract

Acetylcholine is the major excitatory neurotransmitter in the central nervous system of insects. Mutant analysis of the Dα7 nicotinic acetylcholine receptor (nAChR) ofDrosophila shows that it is required for the giant fiber-mediated escape behavior. The Dα7 protein is enriched in the dendrites of the giant fiber, and electrophysiological analysis of the giant fiber circuit showed that sensory input to the giant fiber is disrupted, as is transmission at an identified cholinergic synapse between the peripherally synapsing interneuron and the dorsal lateral muscle motor neuron. Moreover, we found that*gfA^1^,* a mutation identified in a screen for giant fiber defects more than twenty years ago, is an allele of*Dα7*. Therefore, a combination of behavioral, electrophysiological, anatomical, and genetic data indicate an essential role for the Dα7 nAChR in giant fiber-mediated escape in*Drosophila*.

## Abstract 2

The authors integrate behavioral, electrophysiological, anatomical, and genetic data to establish an essential role for the Dα7 nAChR in giant fiber-mediated escape in*Drosophila.*

## Introduction

Nicotinic acetylcholine receptors (nAChRs) mediate fast signaling at most central synapses in the*Drosophila* central nervous system. The*Drosophila* genome contains ten nAChRs, of which three (Dα5, Dα6, and Dα7) are more homologous to vertebrate α7 receptors than to other insect nicotinic receptors [1]. These receptors have unique properties that set them apart from other nicotinic subunits: They can form homomeric channels consisting of only the α subunit, have high permeability to calcium, and desensitize rapidly in the continued presence of agonist [2–4]. Despite the prevalence of acetylcholine in the*Drosophila* central nervous system, to our knowledge so far no studies have analyzed the function of these receptors in vivo. Here we have investigated the role of the Dα7 receptor in behaviorally relevant circuits.

In many invertebrates and vertebrates, intense selection pressure has led to dedicated reflex circuits that continuously monitor the environment for danger and trigger escape behaviors when presented with a specific set of stimuli. These circuits must be able to detect potentially threatening stimuli and respond within a minimal time frame to prevent capture. In dipteran insects the giant fiber system serves this function [5]. The rapid approach of a predator produces an expanding shadow at the retina. Although in wild-type flies stereotypical visual stimuli may be necessary to cause spiking in the giant fiber, rapid dimming of ambient light is sufficient to activate this interneuron in white-eyed mutants [6–8]. One explanation, suggested by Wyman et al. [5], is that because of the screening pigments in the eyes of wild-type flies, only a small number of ommatidia are activated by the light-off stimulus, while in white-eyed mutants the same stimulus can activate a much larger number of ommatidia, hence increasing the stimulus input to the giant fiber.

Two of the cell types that are immediately presynaptic to the giant fiber have been identified anatomically. Visual input arrives via a subtype of the lobula columnar neurons (Lcn neurons in*Drosophila* [9]) called the Col A neurons [10,11]. Additionally, mechanosensory afferents from the antennal chordotonal organs found between the first and second antennal segments also make monosynaptic connections onto the giant fiber. The visual and mechanosensory projections make both electrical and chemical synapses with the giant fiber, but the primary excitatory drive to the giant fiber has been proposed to be mediated exclusively by electrical synapses, while chemical synapses are thought to have an inhibitory role [12]. However, no direct evidence indicates that this is the case, and the transmitter of Col A neurons has not yet been identified. Furthermore, chordotonal organs of the antenna have been shown to be cholinergic [13].

The giant fiber integrates sensory input and activates flight motor neurons in a precise temporal order. Based on electrophysiological and anatomical data from the giant fiber circuit, the following model has been proposed for the sequence of muscle activation underlying the escape jump. First, the tergotrochanteral muscle (TTM) is triggered through electrical synapses, causing the mesothoracic leg to extend and initiate the jump. A second consequence of the activation of this muscle is the compression of the thorax, leading to stretch activation of the dorsal lateral muscle (DLM), which in turn stretches and activates the antagonistic dorsal ventral muscles and causes a self-perpetuating oscillation of the thorax that powers the wings [14]. The timing of DLM activation is delayed relative to TTM activation to precisely control the initiation of flight. The delay in DLM activation is achieved through an interneuron, the peripherally synapsing interneuron (PSI), that is specialized to activate all six DLMs simultaneously via a cholinergic synapse [15].

The DLMs are stretch-activated muscles that, during normal flight, receive nervous system input only once every 20 cycles to prime them with calcium. The central rhythm for this nervous input is generated by synaptic interactions between the dendrites of the DLM motor neurons (DLMmns) [16,17]. However, the rapid start of the flight motor during the escape jump requires synchronized activation of the DLMs, which bypasses the dendritic structure of the DLMmns. Although synchronized DLM activation is an integral part of the escape flight initiation program and is required for flight, it does not directly play a role in jumping. Mutant animals that specifically lose DLMs through degeneration while retaining the TTM are still able to jump, although they are unable to fly [18].

Here, using genetic, immunohistochemical, behavioral and electrophysiological techniques we describe the role of the Dα7 nAChR in the giant fiber mediated escape response of flies.

## Results

### Cloning of the cDNA and Mutational Analysis of Dα7

**Figure 1:**
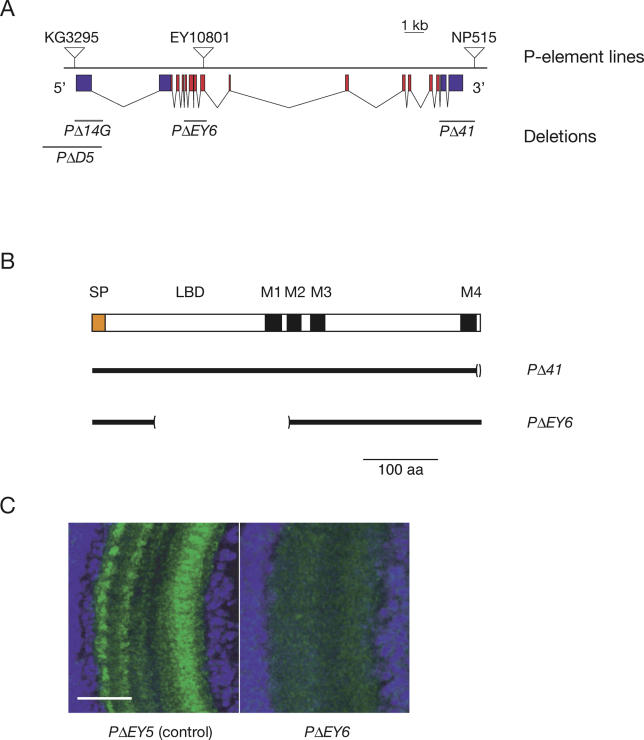
Genomic Structure and Mutational Analysis of the*Dα7* Gene (A) The*Dα7* gene consists of 16 exons. The 5′ and 3′UTRs are drawn in blue, and the ORF is colored red. The insertion sites of the three P-elements are shown above the gene, while the extent of each deletion generated by imprecise excision of the P-elements is depicted below. (B) The structural domains of the Dα7 protein are shown in a schematic representation, and the minimum extent of the lesion associated with the two deletions that remove part of the ORF are indicated by brackets, (), below. Abbreviations: LBD, ligand-binding domain; M1–M4, transmembrane domains 1–4; SP, signal peptide. (C) Immunostaining using an anti-Dα7 antibody is absent in*PΔEY6*. Here representative staining in the medulla is shown. The precise excision*PΔEY5* (left image) was used as a control, and whole-mount of the mutant and control brains were processed together. Scale bar, 20 μm.

To assess the in vivo role of Dα7 we cloned and created mutations in its gene.*Dα7* is located on the X chromosome at cytological band 18C [19]. We generated a full-length cDNA by ligation of two partial cDNAs, RE30878 and GH16126, obtained from the BDGP EST collection. A comparison of the cDNA with the genomic sequence showed that the gene consists of 16 exons that span ∼ 20 kb (Figure 1A). The first two exons are mostly 5′UTR, while the last two exons code for the 3′UTR. Since the protein predicted from our sequence differed from the one recently reported by Lansdell and Millar [20], we performed RT-PCR from two wild-type strains, Canton-S and Hikone-R, and confirmed our protein sequence (Figure S1A). During sequencing we noticed that the protein predicted by our RT-PCR sequence had a substitution of valine for isoleucine 295 when compared with the protein predicted by the published genomic sequence (Figure S1B). This is a consequence of a basepair difference from A in the genomic sequence to a G in the cDNA, indicating the presence of RNA editing, as reported for other nAChRs from*Drosophila* [1]. Sequencing the genomic region from Canton-S flies confirmed this hypothesis.

To create mutations in*Dα7* we identified three P-element insertions: KG3295 maps 25 bp upstream of the 5′UTR, EY10801 maps to exon 8 [21], and NP515 maps 580 bp downstream of the 3′UTR (Figure 1A) [22]. We generated several additional alleles by carrying out imprecise excisions of each P-element. None of the KG3295 excisions disrupted the open reading frame (ORF), since it is approximately 5 kbp upstream of the translation start site. However, we were able to recover two deletions,*Dα7^PΔ14G^* (abbreviated to*PΔ14G*) and*Dα7^PΔD5^ (PΔD5),* that result in a small and a large reduction in protein levels (see below), respectively. The location and the breakpoints of the excisions are shown inFigure 1A. Excisions of NP515 also resulted in a hypomorphic allele (as judged by protein levels, see below),*Dα7^PΔ41^ (PΔ41),* although it lacks the entire 3′UTR and several amino acids from the C terminus (Figure 1B). Imprecise excision of the EY10801 P-element yielded the allele*Dα7^PΔEY6^ (PΔEY6),* which showed loss of almost the entire ligand-binding domain, the transmembrane domain M1, and part of the pore-lining helix M2 (Figure 1B). This deletion also removed a splice site in M2 and hence has a larger lesion than predicted by analysis of the breakpoints. In the remainder of the paper, the precise excision of KG3295,*Dα7^PΔL1^ (PΔL1),* which completely removed the P-element and reverted the insertion site to wild-type sequence, is used as a control for mutants from the KG3295 and NP515 excisions, while the precise excision of EY10801,*Dα7^PΔEY5^ (PΔEY5),* is used as a control for*PΔEY6*.

Using an antibody that we generated against the variable cytoplasmic loop between M3 and M4, we tested for presence of protein in our mutant alleles with immunohistochemistry. The three alleles*PΔ14G, PΔ41,* and*PΔD5* retained signal, with*PΔ14G* showing the strongest staining followed by somewhat weaker staining in*PΔ41,* and still weaker staining in*PΔD5* (unpublished data)*.* As shown for the medulla, a synapse-rich region in the optic lobe (Figure 1C), in*PΔEY6* the staining was reduced to background levels almost everywhere. This antibody recognizes numerous synapses in specific regions throughout the brain and in the ventral ganglion in wild-type animals, most notably in the vicinity of the giant fiber dendrites, where the staining is 10–100 times stronger than anywhere else (see below). However, some synapses in the lamina also stained positively in mutants. These data suggest that the antibody recognizes an epitope on Dα7, that Dα7 is widely expressed (seeFigure S2), and that the antibody also recognizes another epitope whose expression is restricted to the lamina (seeProtocol S1 for antibody expression pattern and discussion of its specificity). Additionally, to identify the cells that express Dα7 protein, we generated an enhancer trap GAL4 line by replacing the KG3295 P-element with a GAL4 P-element using P-element conversion [23]. The staining pattern of this enhancer trap matched the immunostaining observed with the Dα7 antibody (seeFigure S2).

### *Dα7* Mutants Are Defective in a Visually Mediated Escape Behavior

Flies mutant for Dα7 are viable and not readily distinguishable from wild-type flies by cursory observation. To establish whether there are behavioral deficits in flies carrying Dα7 mutations, we tested mutant flies in assays that allowed us to assess the function of brain regions in which Dα7 is expressed (seeProtocol S1).

#### Flight

We tested the flight ability of the mutants in a version of the Sparrow test [24,25]. In this assay, single flies are released from an Eppendorf tube into a 30-cm high cylinder. Good fliers fly horizontally toward the walls of the cylinder and land close to the level at which they were dropped. The lower the fly lands, the worse it is considered at flying. As shown inFigure 2A, we found no significant difference between mutant and control flies.

**Figure 2:**
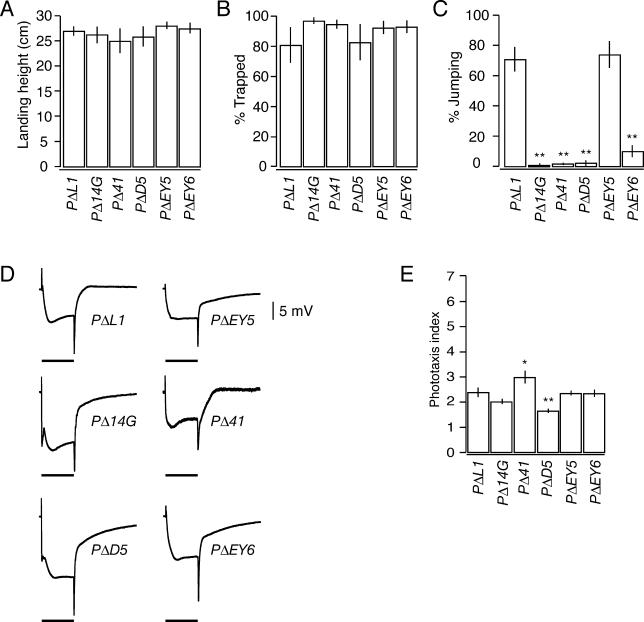
Behavioral Characterization of*Dα7* Mutations The response of flies in a variety of behavioral assays is shown in this figure. The error bars represent SEM. In all assays, the precise excision of P-element line KG3295,*PΔL1,* served as a control for the mutant lines*PΔ14G, PΔ41,* and*PΔD5*. The precise excision of line EY10801,*PΔEY5,* served as a control for*PΔEY6*. (A) Flight test. Flies were individually dropped in a plastic cylinder, and the height at which they landed was recorded. Ten flies were tested for each genotype. (B) Olfactory response. Ten flies were placed in a Petri dish containing traps baited with food, and the number of trapped flies was recorded. Averages were calculated from between eight and ten trials for each genotype. (C) Visually mediated jump assay. Flies were placed in a Petri dish illuminated with green LEDs. A lights-off stimulus was presented by turning off the LEDs for 20 ms. For this assay it was necessary to use white-eyed flies. Since*PΔEY5* and*PΔEY6* are red-eyed, we placed them in a*bw; st* background, which produces white-eyed flies.*p*-Values:*PΔ14G,* 1.9 × 10^−6^ (*n* = 9);*PΔ41,* 1.9 × 10^−6^ (*n* = 11);*PΔD5,* 1.9 × 10^−6^ (*n* = 9);*PΔEY6,* 1.9 × 10^−6^ (*n* = 7). (D) Representative ERGs are shown for control and mutant alleles. We recorded extracellular responses from the eye to 1-s pulses of white light. The timing of the light pulse is shown below each voltage trace. (E) Visual performance was tested using the counter-current assay of Benzer [29].*p*-Values:*PΔ14G,* 0.22 (*n* = 5);*PΔ41,* 0.02 (*n* = 5);*PΔD5,* 0.003 (*n* = 5);*PΔEY6,* 1 (*n* = 5).

#### Olfactory trap assay

The Dα7 receptor is expressed in various elements of the olfactory neuropil, including the antennal lobes and the mushroom bodies. In order to test if loss of the Dα7 receptor resulted in a deficit in olfactory sensitivity, we tested the mutant alleles in the olfactory trap assay that scores the ability of flies to find a source of odor, in this case fly food [26]. As shown inFigure 2B, the mutant flies did not behave differently from control flies.

#### Visually mediated jump

Like many animals, flies have startle reactions that are activated when they sense danger. One well-studied reflex is the giant fiber-mediated response to a sudden change in light levels. We tested the mutant flies in this paradigm to evaluate both visual performance and sensory-motor integration. As shown inFigure 2C, while the two control lines*PΔL1* and*PΔEY5* showed a robust response to a 20-ms pulse of darkness, all of the mutant flies failed to respond (*p* < 0.0001).

#### Visual tests

Since Dα7 is expressed in the optic neuropil, it is possible that the mutant flies are blind and unable to sense light. We, therefore, performed two separate assays of visual system performance by (1) direct electrophysiological assessment of processing at the first visual neuropil, the lamina, with electroretinogram (ERG) recordings [27]; and (2) a different vision-dependent behavior, the phototaxis assay.

The ERG response to a pulse of light consists of three components: the depolarization that corresponds to the response of the photoreceptor to light, the on-transient that is a postsynaptic response of the lamina monopolar cells to photoreceptor depolarization [27], and the off-transient, which is not well understood but may have a cholinergic component [28]. As shown inFigure 2D, all of the mutants responded to light with a depolarization and on- and off-transients.

Since Dα7 is expressed in the optic neuropil, it is possible that the mutant flies are unable either to sense light or to transmit this signal to the locomotor areas. Visual performance was tested in the countercurrent phototaxis assay of Benzer [29] as described by Connolly and Tully [30]. In this assay flies are tested in seven different trials and are sorted into eight classes according to the number of trials in which they walk toward the light source. From these data, a phototaxis index is calculated using a weighted average of the responses in each class. Although two alleles,*PΔ41* and*PΔD5,* showed differences compared to wild-type flies, all mutant alleles moved toward light, indicating that no substantial problem in light sensation can be linked to deletions in*Dα7* (Figure 2E). Taken together, the results from the phototaxis and ERG assays indicate that flies with mutant alleles do not have a generalized defect in basic visual performance.

### *Dα7* Mutants Have a Defect in the Jump Circuit

**Figure 3:**
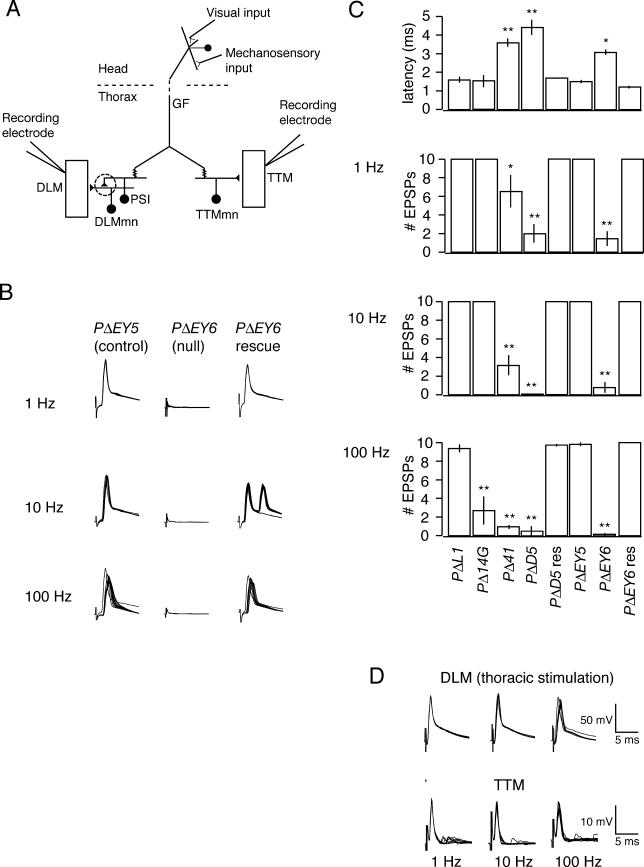
Defective Neurotransmission at the PSI-DLMmn Synapse in*Dα7* Mutants The response of the DLMs to direct activation of the giant fiber is shown. The allele*PΔL1* served as control for*PΔ14G, PΔ41,* and*PΔD5,* while*PΔEY5* served as control for*PΔEY6*. For rescue of the*PΔD5* and*PΔEY6* mutant alleles, experiments were performed in males with genotype*PΔD5/Y; UAS Dα7/OK307 GAL4* and*PΔEY6/Y; UAS Dα7/OK307 GAL4,* respectively. (A) Schematic representation of the giant fiber circuit. Visual and mechanosensory input is transmitted via mixed electrical and chemical synapses to the giant fiber, which carries it to the thoracic ganglion. The TTMmn and PSI neurons are connected via electrical synapses to the giant fiber, and the PSI makes a chemical synapse onto the axon of the DLMmns (encircled). (B) Representative traces of intracellular recordings from DLM muscles for*PΔEY5, PΔEY6,* and*PΔEY6/Y; UAS Dα7/OK307 GAL4*. (C) The response of the mutant and control alleles to giant fiber stimulation is summarized in histograms. The top bar graph shows the average latency of the responses at 1 Hz. The second, third, and fourth bar graphs show the number of responses to ten stimuli at 1 Hz, 10 Hz, and 100 Hz respectively. The*p*-values are as follows. For 1 Hz:*PΔL1, n* = 6;*PΔ14G,* 1 (*n* = 6);*PΔ41,* 0.04 (*n* = 7);*PΔD5,* 2 × 10^−6^ (*n* = 5);*PΔD5/Y; UAS Dα7/OK307 GAL4,* 1 (*n* = 4);*PΔEY5, n* = 5;*PΔEY6,* 2 × 10^−6^ (*n* = 9);*PΔEY6/Y*;*UAS Dα7/OK307 GAL4,* 1 (*n* = 5). For 10 Hz:*PΔL1, n* = 6;*PΔ14G,* 1 (*n* = 6);*PΔ41,* 2 × 10^−6^ (*n* = 7);*PΔD5,* 2 × 10^−6^ (*n* = 5);*PΔD5/Y; UAS Dα7/OK307 GAL4,* 1 (*n* = 4);*PΔEY5, n* = 5;*PΔEY6,* 2 × 10^−6^ (*n* = 9);*PΔEY6/Y; UAS Dα7/OK307 GAL4,* 1 (*n* = 5). For 100 Hz:*PΔL1, n* = 6;*PΔ14G,* 4 × 10^−6^ (*n* = 6);*PΔ41,* 2 × 10^−6^ (*n* = 7);*PΔD5,* 2 × 10^−6^ (*n* = 5);*PΔD5/Y; UAS Dα7/OK307 GAL4,* 0.9 (*n* = 4);*PΔEY5, n* = 5;*PΔEY6,* 2 × 10^−6^ (*n* = 9);*PΔEY6/Y; UAS Dα7/OK307 GAL4,* 0.7 (*n* = 5). All error bars represent SEM. The*p*-values for latency measurements at 1 Hz are:*PΔL1,* (*n* = 5);*PΔ14G,* 1 (*n* = 6);*PΔ41,* 8.6 × 10^−5^ (*n* = 5);*PΔD5,* 3.2 × 10^−5^ (*n* = 2);*PΔD5/Y*;*UAS Dα7/OK307 GAL4,* 1 (*n* = 4);*PΔEY5,* (*n* = 5);*PΔEY6,* 0.02 (*n* = 3);*PΔEY6/Y*;*UAS Dα7/OK307 GAL4,* 1 (*n* = 5). (D) The top panel shows representative intracellular recording from the DLM with direct stimulation of its motor neuron at 1, 10, and 100 Hz. The bottom traces show representative recording from TTM with activation of the giant fiber.

Since the*Dα7* mutant flies had a behavioral defect in the visually mediated jump response but responded normally to light in other visual assays, we tested whether there was a defect in the giant fiber circuit itself. The giant fiber circuit mediates escape behavior in*Drosophila* and is named for the largest interneuron in the fly brain, the giant fiber, which connects the visual neuropil to the flight circuit in the thoracic ganglion (for review see [5]). As shown inFigure 3A, the giant fiber terminates in two branches. One branch makes an electrical connection with the motor neuron of the TTM, or jump muscle, while the other branch makes an electrical connection with an interneuron called the PSI. The PSI in turn makes a chemical synapse with the five motor neurons supplying the six DLMs (Figure 3A). By electrical stimulation through electrodes in the eyes, it is possible to activate the giant fiber directly and assess the synapses that connect the giant fiber to the flight motor neurons by using activation of the flight muscles as a readout [31].

Using this assay we noticed a deficit in the DLM response to giant fiber stimulation that correlated with the strength of the alleles as estimated by protein levels (Figure 3B and3C). The DLMs of wild-type and control flies were able to follow stimulation of the giant fiber at frequencies of up to 100 Hz.*PΔ14G* responded robustly to giant fiber stimulation at frequencies of 1 and 10 Hz, but started to fail when stimulated at 100 Hz.*PΔ41* followed reasonably well at 1 Hz, although it had an abnormally long latency (Figure 3C top panel). At 10 Hz this mutant started to fail, and it had little response at 100 Hz.*PΔD5* showed little response in the DLMs even at a stimulation frequency of 1 Hz, while the null mutant*PΔEY6* did not respond at all at this frequency in most animals (Figure 3B). The defects are specific to the synapses between the PSI and the DLMmn (encircled inFigure 3A), since the TTMs were able to follow the giant fiber stimulation at 100 Hz without any problem in the most severe mutation,*PΔEY6* (Figure 3D). Furthermore, there is no defect at the DLM neuromuscular junction, because direct stimulation of the DLMmn through electrodes in the thorax drove the DLM without failure at 100 Hz (Figure 3D). When we reintroduced the wild-type*Dα7* transgene into the mutant*PΔD5* or*PΔEY6* background by using OK307-GAL4 [32], a driver with expression in several elements of the giant fiber circuit (including the giant fiber, DLMmn, TTMmn and PSI), we were able to fully rescue the activation of the DLM by giant fiber stimulation (Figure 3B and3C). This experiment confirms that the observed defect is due to lesions in the*Dα7* gene. Using these data, we can now put the alleles in the following order according to the strength of the phenotype: wild type =*PΔL1* =*PΔEY5* >*PΔ14G > PΔ41* >*PΔD5* =*PΔEY6.* This order also agrees with the strength of immunostaining.

### *gfA^1^* Is a Dominant Allele of Dα7

**Figure 4:**
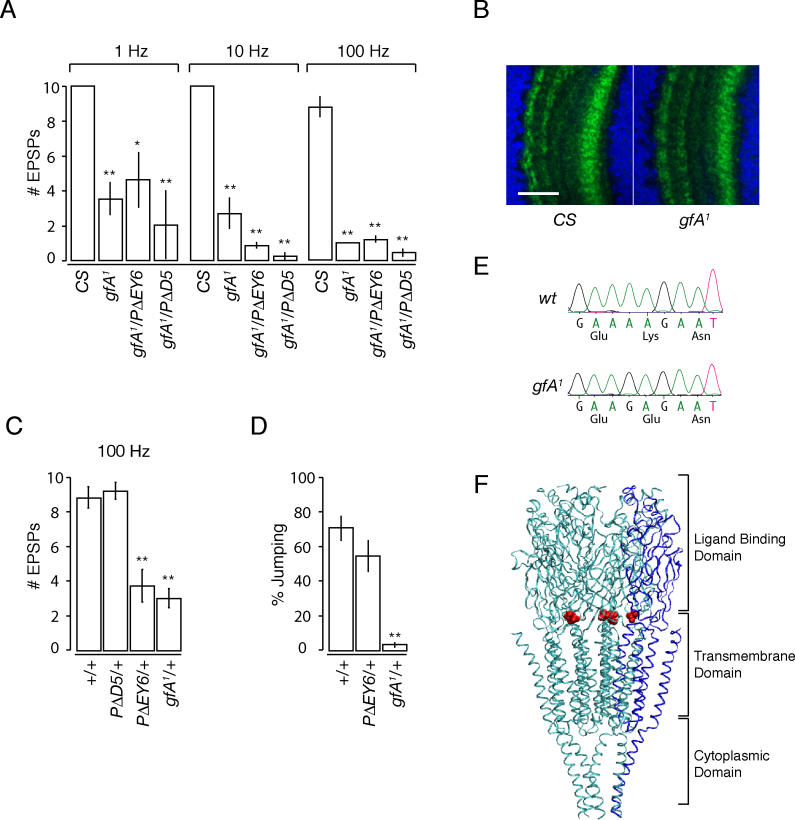
gfA^1^ is a Mutant Allele of*Dα7* (A) The response of transheterozygous animals to giant fiber stimulation at 1, 10, and 100 Hz is shown as a histogram using Canton-S (*CS*) flies as controls.*p*-Values (1 Hz):*CS* (*n* = 6);*gfA^1^,* 0.004 (*n* = 6);*gfA^1^/PΔD5,* 0.0009 (*n* = 4);*gfA^1^/PΔEY6,* 0.02 (*n* = 5).*p*-Values for 10 Hz and 100 Hz were 2.8 × 10^−5^ for all mutant genotypes. (B)*gfA^1^* flies do not show loss of Dα7 protein. Representative staining in the medulla of*CS* and*gfA^1^* flies is shown. Control and mutant flies were processed together for immunohistochemistry. Scale bar: 20 μm. (C)*gfA^1^* and*PΔEY6* show dominant phenotype at the PSI-DLM synapse. Comparison of the response to 100 Hz giant fiber stimulation of*PΔD5, PΔEY6,* and*gfA^1^* in transheterozygous combinations with*CS* flies as controls is shown as a histogram.*p*-Values:*CS* (*n* = 6),*PΔD5*/+*,* 0.99 (*n* = 6);*PΔEY6*/+*,* 0.0002 (*n* = 7);*gfA^1^*/+*,* 0.0001 (*n* = 5). (D)*gfA^1^* flies are dominant in jump behavior. Comparison of jump behavior in*bw; st, PΔEY6/+; bw; st,* and*gfA^1^/+; bw; st* is shown in a histogram.*gfA^1^/+; bw; st* flies fail to jump while*bw; st* and*PΔEY6/+; bw; st* show no significant difference.*p*-Values:*bw; st* (*n* = 10),*PΔEY6/+; bw; st* (*n* = 10), 0.23,*gfA^1^ /+; bw; st*, (*n* = 10), 0.00001. (E) Electropherogram showing basepair change that results in amino acid substitution in*gfA^1^* mutant relative to the*CS* (wt) sequence. (F) Location of mutated residue in*gfA^1^* (shown in red) indicated on the structure of the nAChR fromTorpedo marmorata [35]. This protein consists of five subunits, one of which is depicted in blue, while the others are in cyan. The residue is drawn in van der Waals representation to emphasize its position. This figure was generated using VMD [56].

The defect in the giant fiber in*Dα7* deletion mutants is reminiscent of the phenotype described by Thomas and Wyman [31] for the*gfA^1^* mutants. The phenotype associated with*gfA^1^* was roughly localized by deficiency mapping to interval 18A–D where*Dα7* maps. Like*Dα7* deletion mutants, the DLMs in*gfA^1^* animals followed giant fiber stimulation only at very low frequencies (Figure 4A) [31]. In order to test whether*gfA^1^* is an allele of Dα7, we generated trans-heterozygous flies that contained one copy of*PΔD5* or*PΔEY6* and one copy of*gfA^1^*. As shown inFigure 4A, the DLM in these flies failed to respond to giant fiber stimulation. This complementation test suggests that*gfA^1^* is an allele of*Dα7*.

Since*gfA^1^* does not show any loss of Dα7 protein (Figure 4B), we determined the genetic nature of this mutation. Trans-heterozygous flies*gfA^1^*/+ followed giant fiber stimulation at 1 Hz and 10 Hz. However, at 100 Hz we observed significant failures (Figure 4C). These data show that*gfA^1^* is a weak dominant mutation due to haploinsufficiency or antimorphism. Since homozygous, weak partial loss of function alleles also showed failures at 100 Hz (*PΔ14G,* Figure 3A), the DLM phenotype seems to be very sensitive to gene dosage of Dα7. In order to evaluate this possibility further, we tested trans-heterozygous flies. We observed significant failures at 100 Hz in*PΔEY6/+* but not in*PΔD5/+*. Since*PΔEY6* is a null allele, while*PΔD5* has residual levels of protein, these data suggest that one wild-type copy of*Dα7* is not sufficient for normal function at this synapse when it is heavily challenged.

We obtained further evidence for the antimorphic character of the*gfA^1^* mutation by testing*gfA^1^*/+ flies in the visual jump assay (Figure 4D). Control flies*(bw; st)* jumped 70% of the time (comparable to*PΔEY5; bw; st* flies shown inFigure 2C).*PΔEY6*/+*; bw; st* flies jumped 50% of the time, although this was not significantly different from the control flies. However,*gfA^1^*/+*; bw; st* flies almost always failed to jump. Since*PΔEY6*/+ transheterozygotes performed normally, this experiment provides additional evidence for a dominant-negative character of the*gfA^1^* mutation.

To identify the mutation in*gfA^1^,* we performed RT-PCR and sequenced the mutant gene. We found two nucleotide changes. One of these did not lead to a change in the protein sequence, while the other caused a single amino acid substitution at position 46 from lysine to glutamate (Figure 4E). This amino acid maps to loop 2 of the ligand-binding domain and is implicated in gating by providing a bridge between the ligand-binding domain and the pore-lining transmembrane helix M2 [33,34].Figure 4F shows the location of the analogous amino acid (drawn in red as a van der Waals atomic representation to emphasize the location of the residue) on the structure of the nAChR from the electroplax ofTorpedo marmorata [35]. These data indicate that this single amino-acid change dramatically impairs the activity of Dα7. We propose using the nomenclature*Dα7^gfA1^* for*gfA^1^,* abbreviated*gfA1,* in the rest of the text.

### *gfA1* Mutants Have Reduced Excitatory Drive at the PSI-DLMmn Synapse

**Figure 5:**
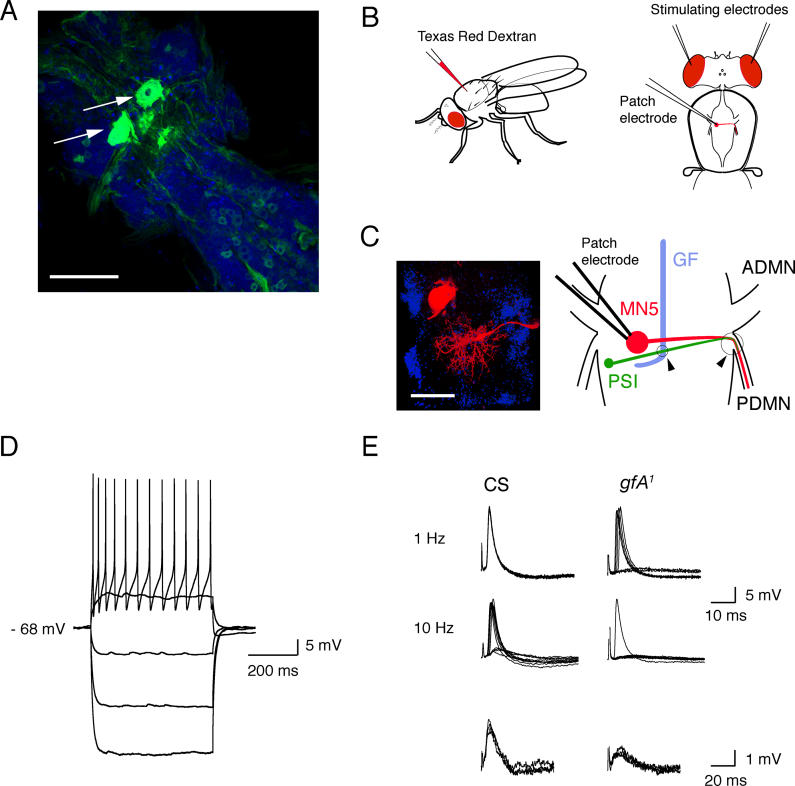
Intracellular Recordings from DLMmn (A) mCD8-GFP driven by*Dα7* GAL4 labels the DLMmns including the dorsal MN5 (arrows). Scale bar: 50 μm. (B) Experimental method for recording from MN5. The fly is injected in DLMs with Texas red dextran, which is then allowed to be transported back to the cell body (left). Scale bar: 50 μm. Whole cell patch clamp recordings are made from the cell body after dissection to remove the asynchronous flight muscles and the gut. The giant fiber was stimulated through insulated tungsten electrodes placed in the eyes. (C) The backfilled MN5 is shown on the left. The organization of the branch of the giant fiber circuit supplying DLMs is depicted on the right. Synapses between the giant fiber and PSI and between the PSI and DLMmn are circled and indicated with arrowheads. Abbreviations: ADMN, anterior dorsal motor nerve; GF, giant fiber; PDMN, posterior dorsal motor nerve. (D) Response of DLMmn to current injection is shown. Current pulses in steps of 200 pA were applied starting at −500 pA. (E) The top tracings show recordings from MN5 in response to 1-Hz giant fiber stimulation. The middle tracings show stimulation at 10 Hz. The bottom tracings show several overlaid EPSPs at higher magnification.

The DLM in the*gfA1* mutant responds to strong electrical stimulation through the eyes, albeit at a lower frequency and with increased latency and jitter when compared to control animals. One possible explanation is that, due to changes in the nAChR properties, the excitatory postsynaptic potential (EPSP) generated at the PSI-DLMmn synapse in*gfA^1^* mutant animals is faster and decays so rapidly that it is not able to trigger an action potential, similar to the effect of nAChR mutations in fast channel myasthenic syndrome [36]. An alternative possibility is that the amplitude of the EPSP is reduced in*gfA1* mutants so that it is insufficient to drive the cell to threshold. In order to directly test this possibility, we developed a novel preparation for performing whole-cell patch clamp recordings from one of the DLM motor neurons, MN5, that supplies two muscles, DLMa and b. MN5 is located on the dorsal surface of the thoracic ganglion and is prominently labeled with CD8-GFP under the control of Dα7-GAL4 (Figure 5A).

The basic methodology for recording from these neurons is depicted inFigure 5B. In spite of their large size and characteristic location, MN5 neurons are not readily detectable under Nomarski optics. Therefore, we backfilled these neurons by injecting Texas red dextran into the thorax in the vicinity of the DLMa/b muscles and waiting 18–22 h to allow transport of the dye into the motor neuron. To access these neurons for recording, we removed the indirect flight muscles and the gut to expose the thoracic ganglion. As shown inFigure 5C, following the backfill procedure, the cell body of the motor neuron was clearly visible, as were the primary neurite and some of the dendritic structure. By locally applying protease, we were able to gain access to the cell body for whole cell patch recording without damaging the giant fiber, which was activated through insulated tungsten stimulating electrodes placed in the eyes. Protease treatment can affect electrophysiological measurements by cleaving molecules such as certain ion channels. While we cannot rule out these effects in our experiments, neurons in the DLM branch of the giant fiber circuit appeared healthy, as judged by the input resistance of MN5 as well as its ability to generate action potentials in response to current injection and EPSPs in response to giant fiber stimulation.

We recorded potentials from these cells using whole-cell patch clamp. Since no differences in basic passive properties of the cells were found between Canton-S controls and mutant flies, the average of the combined datasets is reported. In keeping with the large size of these cells, the capacitance of these cells was 43 ± 2 pF (*n* = 22), and their average input resistance was 102 ± 12 MΩ (*n* = 22). Unlike other insect neurons, current injection into MN5 cell bodies can generate large action potentials of 50 to 60 mV (Figure 5D). In a few preparations we observed spontaneous rhythmic activity at ∼ 1 Hz consisting of plateau potentials with large spikes, suggesting that these cells receive patterned input close to the cell body and are able to generate action potentials close to this site (unpublished data).

Since the primary role of the giant fiber circuit is to initiate escape behavior, the PSI bypasses the entire dendritic structure of MN5 and makes multiple synaptic contacts that span 50 μm and follow the axons out into the posterior dorsal motor nerve [37,38]. This specialization minimizes the delay to DLM activation by generating a suprathreshold EPSP distally in the axon, which leads to a spike that travels rapidly to the DLM. In wild-type animals, when we activated the giant fiber at 1 Hz, the action potential generated at the distal PSI-DLMmn synapse on the axon appeared small and broad in somatic recordings (Figure 5E). This differed considerably from the narrow, large action potential evoked in the soma by current injection (Figure 5D), suggesting that the peripherally generated action potential fails to propagate antidromically into the dendrite and therefore is filtered by the passive membrane properties of the dendritic and/or axonal structure (Figure 5E, top). As we increased the frequency of eye stimulation, the action potential failed to fire, revealing an underlying EPSP (Figure 5E middle). The EPSPs are shown at a higher magnification in the bottom panel ofFigure 5E to emphasize their amplitude and shape.

In order to measure the amplitude of the EPSP, we used three different strategies that all gave approximately the same values when compared in the same preparation. The first was to directly measure the amplitude of the EPSP at high frequencies when the spike fails. The second was to measure the amplitude to the inflection point where the spike takes off from the EPSP in cases where it is clearly visible. The third was to trigger an action potential by current injection in the MN5 soma just before giant fiber activation to force an absolute refractory period at the PSI-DLMmn synapse, causing the spike to fail thereby revealing the EPSP [39]. The mean amplitude of the synaptic potential underlying the spike measured at the soma using these three methods was 4.71 ± 0.35 mV (*n* = 10) in wild-type animals.

In*gfA1^1^* animals, we observed considerable failures even at 1 Hz, similar to what we observed in muscle recordings from the intact animal (Figure 5E). The spikes showed a considerable amount of jitter and began at different points of the EPSP. The underlying EPSP is significantly smaller than the EPSP generated in wild-type animals (*p* = 0.00001, Student's t-test), with a mean amplitude of 1.5 ± 0.32 mV (*n* = 7). However, while smaller, the EPSP in*gfA1* animals followed without failure and did not show significant changes in shape or jitter. This suggests that the inability of the DLMmn to follow and the increase in jitter in*gfA1* mutants is most likely due to reduced synaptic drive at the PSI-DLM synapse. This observation also rules out the possibility that a slow, alternative pathway is being activated in*gfA1* mutants during electrical stimulation through the eyes.

### Dα7 Is Present in the Dendrites of the Giant Fiber

The electrophysiological results presented so far show a defect downstream of the giant fiber but do not explain the behavioral data. Indeed, as shown inFigure 2C, all the*Dα7* mutants failed to jump in response to a “lights off” stimulus, yet the branch of the giant fiber neuron synapsing with the jump muscle (i.e., TTM) motor neuron is intact and functional in all the mutants tested. Since the DLM has not been implicated in jumping in*Drosophila* [18], we postulated that perhaps there is an additional defect in the giant fiber circuit. This defect should be prior to or at the input of the giant fiber, such that it renders it unresponsive to visual stimuli in mutant animals.

**Figure 6:**
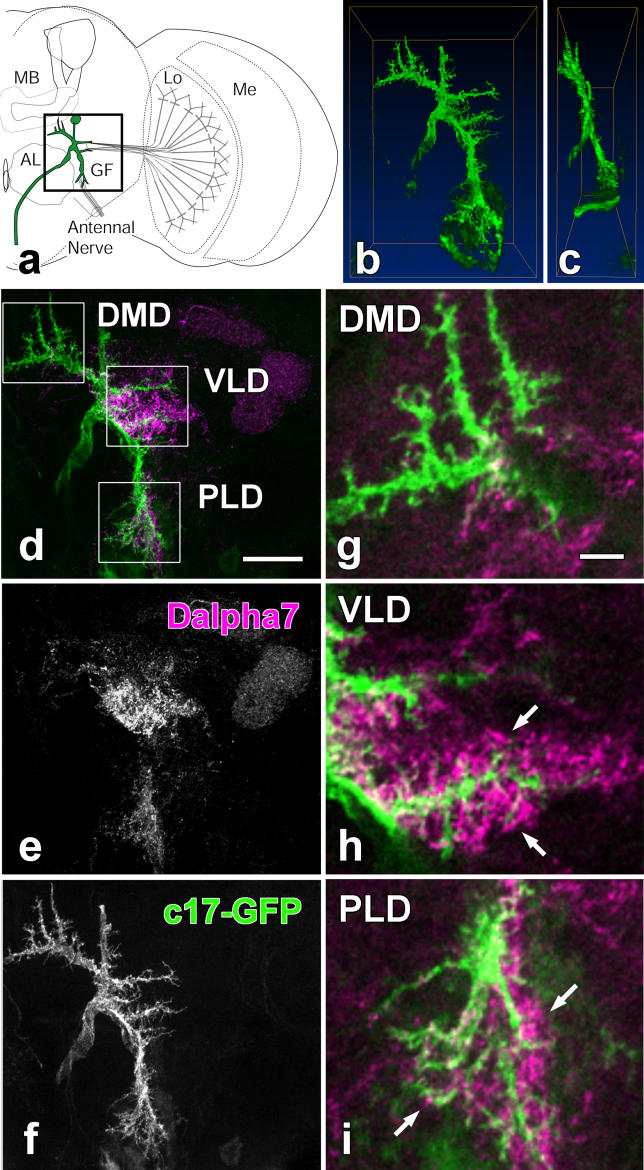
Expression of Dα7 in the Giant Fiber (A) Schematic of the*Drosophila* brain showing the location of the giant fiber in relation to other brain regions. The inputs to the giant fiber dendrites from the lobula and antennal nerve are also shown. Abbreviations: AL, antennal lobe; GF, giant fiber; Lo, lobula; MB, mushroom body; Me, medulla. (B) Frontal view of the giant fiber dendrites viewed from the posterior. (C) Sagittal view of the giant fiber dendrites showing the anterior-posterior position of the various dendritic branches. (D–F) Colocalization of Dα7 immunostaining in the dendrites of the giant fiber. Projection of the giant fiber dendrite (green) labeled with CD8-GFP driven by the c17 GAL4 driver and Dα7 staining (magenta) onto the same plane is shown in (D). Single channels of antibody staining (E) and GFP (F) are shown. The three major subdivisions of the dendrites are boxed and labeled according to the terminology of Allen et al. 1998 [32]. Abbreviations: DMD, dorsal medial dendrite; VLD, ventral lateral dendrite; PLD, posterior lateral dendrite. (G–I) Magnified views of the three dendritic fields shown in (D). Images of DMD and PLD shown in (G) and (I), respectively, are projections of eight confocal slices for a total of 4 μm thickness. The image of VLD shown in (H) is a single confocal section of 500 nm. Arrows show Dα7 staining decorating giant fiber dendrites. Bounding box in (B) and (C): 140 × 71 × 20 μm. Scale bar: 20 μm (D); 5 μm (G).

The dipteran giant fiber receives mechanosensory input via the Johnston's organ in the antenna [6,8,12] and visual input via the photoreceptors and optic lobes. The mechanosensory neurons connect directly with the posterior lateral dendrite (PLD) of the giant fiber (Figure 6A–6C), while the visual input has to travel through at least four synapses to reach the giant fiber [6,40]. Of these, the lobula columnar neurons are immediately presynaptic to the giant fiber neuron and make connections onto the ventral lateral dendrites (VLDs) (Figure 6A–6C) [6].

Dα7 is localized to the dendritic tree of the giant fiber neuron (Figure 6D–6F). Projections of confocal images of the dendrites show that the strongest Dα7 staining in the brain indeed colocalizes with the VLD and PLD dendritic arbors. By contrast, the dorsal medial dendrite, where the giant commissural interneurons synapse [32] is not labeled (Figure 6D and6G). High-resolution scans of the VLD and the PLD (Figure 6H and6I) show a punctate pattern of Dα7 staining that decorates the GFP-labeled dendrites. While the colocalization with antibody staining is almost complete for the PLD, the anti-Dα7 label is present in a broader field relative to the VLD. This may be explained by the presence of other dendritic trees of large neurons in precisely this area of the brain [8] or smaller dendritic spines that were not picked out by the scan either because of their small size or because they were not sufficiently bright.

### Dα7 Mediates Synaptic Transmission at the Dendrites of the Giant Fiber

**Figure 7:**
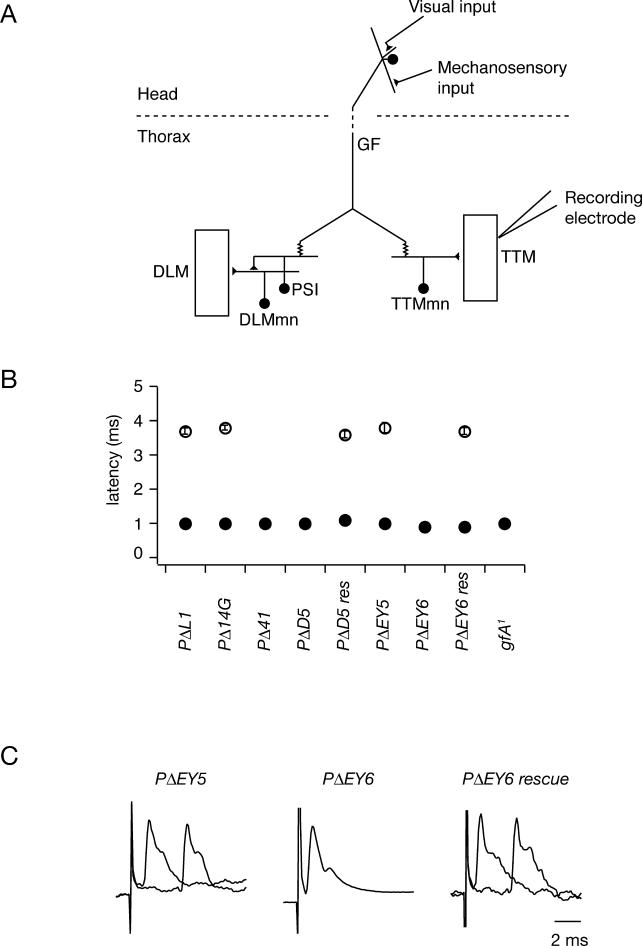
Long-Latency Response of TTM to Giant Fiber Stimulation (A) Recording configuration. The giant fiber was stimulated via tungsten electrodes in the eyes, and extracellular potentials were recorded from the TTM. (B) Average long- and short-latency response of TTM to giant fiber stimulation in different genotypes.*PΔD5 rescue* and*PΔEY6 rescue* had genotypes*PΔD5/Y; UAS Dα7/c17 GAL4* and*PΔEY6/Y; UAS Dα7/c17 GAL4,* respectively. The success rate of long-latency stimulation was as follows:*PΔL1* 8/10,*PΔ14G* 2/4,*PΔ41* 0/5,*PΔD5* 0/6,*PΔD5/Y; UAS Dα7/c17 GAL4* 2/2,*PΔEY5* 4/6,*PΔEY6* 0/4,*PΔEY6/Y; UAS Dα7/c17 GAL4* 3/3,*gfA^1^* 0/3. (C) Representative traces showing the presence of short- and long-latency responses.*PΔEY5* and*PΔEY6/Y; UAS Dα7/c17 GAL4* showed both short- and long-latency TTM responses, but the long-latency response was absent in*PΔEY6*.

Since Dα7 is localized to the dendrites of the giant fiber, we wished to test whether loss of Dα7 leads to loss of transmission at these synapses. In order to test this hypothesis we took advantage of the observation that, in contrast to strong stimulation, which directly activates the giant fiber, low-strength electrical stimulation activates the elements presynaptic to the giant fiber, which in turn activate the giant fiber [41]. We therefore recorded from the TTM and stimulated the eyes at low strength (Figure 7A). As shown inFigure 7B and7C, low-strength stimulation, which activates the presynaptic elements of the giant fiber, results in a significantly longer latency to activation of the TTM (3.5–4.0 ms) compared with high-strength stimulation, which activates the giant fiber directly (∼ 1 ms). These values are comparable to long-latency values reported in the literature, which vary from ∼ 3–4.2 ms [42–45]. Since the TTMmn is electrically coupled to the giant fiber, activation of the TTM serves as a readout of giant fiber activity. When we tested the*Dα7* mutant alleles in this paradigm, we discovered that only the weakest allele,*PΔ14G,* showed a long-latency response (upon low-strength stimulation), as well as a short-latency response (upon high-strength stimulation), while all other mutant alleles responded only with short latency (see Discussion for*PΔ14G*). These data suggest that there is a defect in synaptic transmission somewhere in the pathway either upstream of the giant fiber or at the input of the giant fiber itself. In order to distinguish between these possibilities, we carried out a rescue experiment using a giant fiber-specific driver, c17-GAL4 [46]. Reintroducing a wild-type copy of*Dα7* into the giant fiber using this driver completely rescued the long-latency response, indicating that the lack of Dα7 in the giant fiber itself is responsible for the mutant phenotype. Furthermore, since absence of Dα7 prevents activation of the giant fiber, these experiments provide strong evidence that the defect in jump behavior is due to the loss of Dα7 in the giant fiber neuron.

## Discussion

Using anatomical, behavioral and physiological techniques to analyze mutant alleles of a nAChR, Dα7, we show that this receptor is essential for the giant fiber-mediated escape response in*Drosophila*. Flies with mutations in*Dα7* do not jump in response to a “lights off” stimulus. Using electrophysiological and anatomical evidence, we show that the visual and mechanosensory inputs on the dendrites of the giant fiber are cholinergic, and that loss of Dα7 in the giant fiber is responsible for the behavioral deficit in the visually mediated escape response. Furthermore, the cholinergic synapse between the PSI and DLMmn is defective in*Dα7* mutants. Finally, we find that*gfA^1^,* a previously molecularly uncharacterized mutant isolated in a behavioral screen for giant fiber defects, is a missense mutant of*Dα7* that shows diminished EPSPs at the PSI-DLMmn synapse.

The*gfA1* mutant phenotype is caused by the amino acid substitution K46E in loop 2 of the ligand binding domain of Dα7. Interestingly, although mutations in loop 2 of the ligand binding domain of nAChRs have been the focus of a number of structure-function studies in recent years (for review see [34]), this is the first mutant in this domain that has been linked to a genetic phenotype. This region has been implicated in coupling ligand binding to gating in a number of cys-loop receptors that include nAChRs, GABA receptors, and glycine receptors. Charge reversal mutations in K46 of bovine α7 nAChRs, the homolog of Dα7K46, show diminished responses to acetylcholine and can act in a dominant-negative fashion when coexpressed with wild-type receptors [47], similar to what we observed for the*gfA1* mutation. Our data show that the charge of Dα7K46 is critical to the functioning of this receptor and hence synapses mediated by Dα7 containing nAChRs.

The giant fiber circuit has been a model for central circuits in*Drosophila* and has been studied in some detail using elegant experiments that revealed significant details about the circuit in the intact fly. The giant fiber output in the thorax activates at least three pathways: the TTM motor neuron via electrical synapses, the DLM motor neuron via an interneuron (PSI) that is itself electrically coupled to the giant fiber, and the tibial levator muscle motor neuron through a novel pathway that is not well characterized [42]. We show that the PSI-DLMmn synapse, which was previously suggested to be cholinergic [15], is mediated by the Dα7 nAChR. Wyman et al. [5] showed that the*gfA1* mutation affects this synapse. However,*gfA1* was identified in a screen for behavioral defects in the giant fiber-mediated jump response [31]. Since it is known that flies can jump even in the absence of the DLMs [18], the behavioral defect of*gfA1* cannot be explained by the physiological defect at the PSI-DLMmn synapse.

Since the TTM pathway downstream of the giant fiber is not affected by the*Dα7* mutations, a more likely explanation is that the giant fiber is not activated by visual input in*Dα7* mutant animals. We show that this is indeed the case. In most deletion mutations of*Dα7,* the giant fiber did not respond to electrical stimulation of its presynaptic components, and this phenotype could be rescued by overexpressing a transgene coding for*Dα7* specifically in the giant fiber. These data indicate that the behavioral defect in the visually mediated jump in most deletion mutants of*Dα7* can be explained by a failure of activation of the giant fiber. The sole exception is the weak hypomorphic mutation*PΔ14G;* the affected animals respond to electrical stimulation of the elements presynaptic to the giant fiber, and yet fail to jump in response to a “lights off” stimulus. One possible explanation is that electrical stimulation through the eyes may activate both the mechanosensory and visual inputs to the giant fiber, while the behavioral assay relies on a strictly visual input. Therefore, activation by presynaptic electrical stimulation of the giant fiber in*PΔ14G* mutants may reflect the simultaneous activation of neurons presynaptic to the giant fiber that does not occur during normal visual system function.

Our rescue of the long-latency component by expression of a*Dα7* transgene in the giant fiber shows that the chemical synapses onto the giant fiber are cholinergic, and this component provides a significant proportion of the synaptic drive. The sensory projections to the giant fiber in three different dipteran species,*Drosophila* [12],*Musca* [6,12], and*Calliphora* [8,12], make mixed chemical and electrical synapses onto specific dendrites of the giant fiber: The mechanosensory projection synapses onto the PLD, while the lobula projection synapses onto the VLD. Strausfeld and Bassemir [12] hypothesized that the electrical synapses from the Col A and mechanosensory projections provide the excitatory drive to the giant fiber, while the chemical component is inhibitory. Our data clearly show that chemical synapses mediating visual and mechanosensory inputs onto the giant fiber are excitatory in nature and play an essential role in the giant fiber-mediated jump response.

It is probable that additional behavioral defects are present in these mutants that were missed because we performed only a limited number of assays. It is nevertheless surprising, given the widespread expression of this receptor, that the mutant animals did not show many obvious defects and were relatively healthy. One possible explanation is that the large number of nicotinic subunits can functionally compensate for each other. Although all ten nAChRs from*Drosophila* have been cloned, a detailed description of localization exists for only two of these proteins, ALS and ARD, which colocalize in most regions of the brain [48]. Although these two receptors are localized to many of the same regions as the Dα7 protein, analysis of specific neuropils showed some differences in expression patterns. Furthermore, although a number of screens have been carried out in*Drosophila* to find genes implicated in behavior, the only nicotinic receptor that has so far been isolated to our knowledge is*gfA*. Given that ACh is considered the primary excitatory neurotransmitter in the central nervous system of insects, there may be much functional redundancy among the receptors, and only in the few instances when a synapse relies mostly or entirely on a particular nicotinic receptor, such as the visual and mechanosensory inputs on the dendrites of the giant fiber or the PSI-DLM synapse, is it possible to see a phenotype. Functional redundancy has also been observed in nAChR mutants in vertebrates, where some phenotypes are observed only when multiple nicotinic receptors are deleted [49,50]. It is remarkable that redundancy does not extend to the circuit underlying one of the most important behaviors for the day-to-day survival of the fly, the giant fiber-mediated escape circuit. This fact suggests that particular properties of Dα7 may be selected for during evolution to endow certain qualities to the circuit. Further characterization of the biophysical properties of Dα7 both in vivo and in vitro should shed some light on how specializations at the synaptic level are implemented in the choice of neurotransmitter receptor.

## Materials and Methods

### Cloning of Dα7

In order to obtain the full-length cDNA for Dα7, we scanned the BDGP EST database and found four clones that mapped 5′ of the predicted gene for Dα7. We sequenced the largest of these clones, RE30878, and found that it had a small deletion that resulted in an early stop codon and a truncated protein. A second EST, GH16126, did not have this deletion but was truncated further 3′. Making use of a common unique Kpn1 site, we were able to construct a full-length cDNA. The largest ORF predicted from the cDNA has a length of 1,683 bp. However, identifying the Kozak [51] sequence and signal peptide [52], we predict that the translated ORF is 1,563 bp in length, with 5′ and 3′UTRs of 1,205 and 1,056 bp, respectively. This ORF encodes a protein of 502 amino acids (excluding the putative signal peptide of 18 amino acids) with a predicted molecular weight of 57 kDa.

For reverse transcriptase PCR, total RNA was extracted from 30 flies using Trizol reagent (Invitrogen, Carlsbad, California, United States). The reverse transcriptase reaction was carried out using SuperScript II reverse transcriptase (Stratagene, La Jolla, California, United States) primed with poly-dT primers. The ORF was amplified using*Pfu* polymerase and cloned into Blunt-TOPO PCR vectors and sequenced. To establish the mutation in*gfA^1^,* we directly sequenced the PCR product.

### P-element excision

P-element lines*y* KG3295,*yw* NP515, and*yw* EY10801 were crossed to*Ly/Δ2–3 TM3, Sb* to generate excisions. Mutant lines were screened using PCR across the P-element insertion site from pooled genomic DNA. Subsequently, individual stocks from positive pools were screened to isolate mutant lines.

Upon screening an initial 250 excision lines generated from KG3295, we discovered a second site insertion on the X chromosome in the furrowed gene*(fw)*. We removed the insertion in*fw* by recombining against the multiply marked stock*y sn v g f* and recovered recombinants carrying*y sn v g* and not*f,* to make sure that the mutation in*fw* (which maps between*v* and*g*) was lost. To ensure the loss of*fw* we carried out a PCR over the*fw* locus and found no P-element insertion. Subsequently we removed the markers*sn v g* by recombining against*yw* and recovering recombinants that carried*y* ^+^ *w* ^+^ and did not carry any recessive markers. Using this cleaned KG3295 line we carried out an additional 500 excisions. One line from the initial KG3295 excision,*PΔ14G,* was cleaned of the*fw* mutation using a similar strategy.

A total of 500 independent excisions lines each were screened for NP515 and EY10801. Wild-type*y* and*w* genes were reintroduced into*PΔEY5* and*PΔEY6* by recombination against the deficiency JA27 that uncovers the cytological region 18A–D containing the*Dα7* locus.

### Rescue construct

Rescue of the mutant phenotype was carried out using a UAS transgene. The open reading frame of*Dα7* was amplified using*Pfu* polymerase with primers 5′-ACC CAG AGA TCT ATC CAT GAG CTT CCC ACA AC-3′ and 5′-TAT TAT GCG GCC GCC TTC GCT TAC GGG AAA ATG A-3′ with engineered BglII and NotI sites, respectively, which were used to clone the PCR product into the multicloning site of the vector pUAST [53].

### Immunohistochemistry

DNA coding for the cytoplasmic loop between M3 and M4 was amplified with*Pfu* polymerase using primers 5′-CGA CCA GAA TTC ACG CAT GAA ATG AGT GAA TGG-3′ and 5′-GAG ACG GAG CTC TTA GGC AGC AAA TTT CCA ATC T-3′ with engineered EcoRI and XhoI restriction sites that were then used to ligate the PCR product in frame into the pET28a vector (Novagen, Madison, Wisconsin, United States) to generate a His-Tag fusion protein. A rat antibody was raised against this fusion protein (Cocalico, Reamstown, Pennsylvania, United States) and was used at dilutions of 1:2,000 for immunohistochemistry. Optimal staining was obtained when dissected brains or ventral nerve cords were fixed in formalin acetic acid alcohol fixative or 4% formaldehyde in PBS for less than 10 min.

To determine staining intensity levels in mutants, wild-type and mutant specimens were dissected together, and the brains of the mutant animals were split in halves. The brains were subsequently fixed and stained in the same tube, mounted on the same slide, and scanned with the same settings on the confocal microscope.

### Dα7-GAL4

An enhancer trap GAL 4 line was generated by P-element conversion [23] of the KG3295 P-element, which is located just 25 bp upstream of the transcription start site of the*Dα7* gene. A pGawB P-element from a donor on the third chromosome was mobilized in the presence of KG3295 using*Ly/Δ2–3 TM3, Sb* and conversion events were screened by loss of the*y* marker but retention of X-linked red eye color indicating an excision of the SUPor-P P-element of KG3295 and presence of pGawB on the X chromosome. Positive lines were crossed to UAS-mCD8-GFP and the expression pattern of GFP was compared to the anti-Dα7 staining pattern. Insertion in the*Dα7* locus was confirmed by PCR and sequencing.

### Behavioral assays

We tested control and mutant flies in the behavioral assays described below. For all tests the flies were between 2 and 7 d old. Statistical significance was tested with one-way ANOVA using*PΔL1* or*PΔEY5* as controls.

The flight assay was performed according to the method of Drummond et al. [24] as modified by Nelson et al. [25] Briefly, we aspirated single flies into an Eppendorf tube that had its tip sliced off to leave an opening of approximately 3 mm. The fly was then tapped from the tube through a hole in the lid of a cylinder with inner diameter of 70 mm and length 300 mm. which was divided into 12 zones of 25 mm each. The zone in which the fly landed was noted for each fly and used to estimate the landing height. At least ten separate flies were used for each genotype, and the results were averaged. In a few cases flies failed to fly at all and were not used as part of the analysis, since they may have been damaged during handling.

The olfactory trap assay of Woodard et al. [26] was used to test for olfactory defects. Approximately ten cold-anesthetized flies were placed in a Petri dish containing 1% agarose along with a trap containing fly food as described. The number of flies in the trap were counted and expressed as a percentage of the total number of flies per Petri dish. At least eight trials were conducted for each genotype.

Since the visually mediated jump assay works only with white-eyed flies [5], and*PΔEY5* and*PΔEY6* lines are in a*w* ^+^ background, we generated white-eyed*PΔEY5; bw; st* and*PΔEY6; bw; st* flies by crossing and backcrossing males to*C(1) DX; bw; st* flies. Flies were individually aspirated into a 60 × 15 mm Petri dish covered on the bottom and sides with vellum. This Petri dish was placed inside a 100 × 15 mm Petri dish illuminated by four green light-emitting diodes (LEDs; peak wavelength 572 nm) placed at 90° to each other. A “lights off” stimulus was provided by turning off the LEDs for 20 ms using a simple custom device driven by a TTL signal from a pulse generator.

Flies were tested for phototaxis in the countercurrent apparatus of Benzer [29] as described by Connolly and Tully [30]. Groups of 20 flies were given a choice between a vial facing a light source provided by a fluorescent lamp and a vial facing away from the light source. Each fly was given the choice eight times and separated into seven groups according to the number of times they moved toward the light source. A phototaxis index was calculated using the equation (1/*N*) Σ*_i_* *i* •*n_i_* where*i* is the fraction number,*n_i_* is the number of flies in that fraction, and*N* is the total number of flies tested.

### In vivo electrophysiological assays

For all assays, flies were anesthetized with either exposure to CO_2_ or cooling on ice for 5 min, and glued to a glass slide using Elmer's school glue gel.

Electroretinograms were recorded between a microelectrode inserted just below the surface of the eye and a reference electrode in the thorax with an Axopatch 200B amplifier. The light stimulus was provided by a bright white LED driven directly by a 1-s TTL pulse from the digital out port of a Digidata 1322 interface (Axon Instruments, Union City, California, United States) controlled by Axograph 4.9 software. A 150-Ω resistor was placed in series with the LED to limit the maximum current.

To assay the giant fiber system, intracellular recordings were made from DLM 45a using the stereotaxic map of Levine and Hughes [54] using glass microelectrodes filled with 3 M potassium acetate and 0.1 M KCl with an Axopatch 200B amplifier. The giant fiber was electrically stimulated through tungsten electrodes placed in the eyes. In most control flies, the threshold of evoking a high-threshold response was ∼10–15 V with a 100-μsec pulse. Since very large stimuli could activate the DLM motor neurons directly (as judged by very short latency responses), in most cases we kept the stimulus amplitude constant at 30 V.

To evaluate the long-latency response of TTMs, recordings were made using etched tungsten electrodes with an A-M Systems 1800 extracellular amplifier (A-M Systems, Carlsborg, Washington, United States). 10-μs voltage pulses were applied across tungsten electrodes inserted just under the cuticle of the eyes. The voltage was increased in 0.5-V increments every 10 s to minimize habituation [43]. Even with these precautions, in a few cases we were not able to record the long-latency response in wild-type animals. The success rate for each genotype is given in the legend forFigure 6.

### Motor neuron electrophysiology

Control and mutant animals were anesthetized with CO_2_ and the DLM muscles injected with 3,000 MW Texas red dextran solution (5% w/v in water) to backfill the DLM motor neuron. The flies were then allowed to recover for 15–20 h before further experiments.

After clipping off the wings and legs, cold-anesthetized flies were attached ventrally to a glass coverslip using low melting point wax (Paraplast). The thorax was opened dorsally using a 30-gauge hypodermic needle, and the indirect flight muscles and gut were removed to reveal the thoracic ganglion. The body walls were then cut away to allow access for the recording electrode.

The MN5 motor neurons were visualized, under constant perfusion, with a 40× water immersion objective and unambiguously identified by Texas red fluorescence and morphology. The neurolemma was disrupted around the cell body using 0.5% protease (type XIV, Sigma, St. Louis, Missouri, United States) applied through a micropipette as described by Baines and Bate [55]. Fire-polished electrodes made from thin-walled borosilicate glass (3–5 MΩ) were used to patch onto the cell body of MN5. Seals of 2–10 GΩ formed readily, and whole-cell access was achieved with light suction or using the zap function of the Axopatch 200B. Whole-cell capacitance was compensated, and access resistance was generally kept below 20 MΩ. Diffusion of carboxyfluorescein from the patch electrode to the cell unambiguously identified the recorded cell by colocalization of red and green fluorescence. A constant current command was delivered in current clamp mode to keep the voltage at −74 mV to reduce the spontaneous activity of the neuron to that observed in cell-attached mode. The giant fiber was stimulated through insulated tungsten electrodes placed in the eyes. In some cases, stimulation was provided via a suction electrode on the neck connective.

The extracellular solution was composed of (in mM) 120 NaCl, 2.5 KCl, 2 CaCl_2_, 26 NaHCO_3_, 6 MgCl_2_, 5 trehalose, 10 TES, and 15 glucose, and was bubbled continuously with a mixture of 95% O_2_ and 5% CO_2_. The intracellular pipette solution consisted of (in mM) 144 potassium gluconate, 10 HEPES, 1 EGTA, and 3 MgCl_2_. Liquid junction potentials are taken into account for reporting of membrane potentials.

### Statistical tests

Except where indicated, we used a one-way ANOVA to test for significance. Pairwise comparisons were done with Dunnett's test using*PΔL1* or*PΔEY5* as controls. In all figures significance is indicated by * (α = 0.05) or ** (α = 0.01).*p*-Values are stated in the figure legends along with the sample numbers. All reported errors are standard error of the mean (SEM).

## Supporting Information

Figure S1 Features of the Dα7 Protein Sequence (A) Dα7 protein sequence. The sequence for Dα7 obtained in this study (top) is compared with the sequence published by Lansdell and Millar [20] (bottom). Differences are highlighted in red. The start of the coding region is based on analysis of the Kozak consensus sequences for*Drosophila* and signal peptide prediction. The edited amino acid valine 295 is highlighted in blue. The putative extents of the signal peptide (SP), and transmembrane domains (M1–M4) are indicated below the sequence.
(B) Dα7 protein is edited. The genomic DNA and cDNA sequence of the edited region in M4 is shown.
(519 KB PDF).

Figure S2 Expression Pattern of Dα7 The photomicrographs on the left (A, C, E, and G) show immunohistochemical localization of Dα7 in Canton-S flies using an antibody generated against the variable cytoplasmic loop between the M3 and M4 transmembrane domains. The photomicrographs on the right (B, D, F, and H) show mCD8-GFP expression under the control of the*Dα7*-GAL4 enhancer trap line. The top four photomicrographs (A, B, C, and D) show localization in the central brain. The next two photomicrographs show localization in the optic lobes (E, F) and the bottom two photomicrographs show localization in the ventral nerve cord (G and H). Particular regions of the central nervous system are labeled to show the similarity in the structures labeled using the two methods. Abbreviations: al, antennal lobe; aot, anterior optic tubercle; cx, calyx of the mushroom body; dlm, DLM motor neuron cell bodies; lc, lobula complex; me, medulla; of, optic foci. Scale bars: 50 μm.
(3.7 MB TIF).

Protocol S1 Expression of Dα7 in the Fly Central Nervous System Additional details of Dα7 localization in different parts of the*Drosophila* central nervous systems are discussed.
(48 KB DOC).

## Abbreviations

DLMmn: dorsal lateral muscle motor neuron
EPSP: excitatory postsynaptic potential
ERG: electroretinogram
LED: light-emitting diode
nAChR: nicotinic acetylcholine receptor
ORF: open reading frame
PLD: posterior lateral dendrite
PSI: peripherally synapsing interneuron
SEM: standard error of the mean
TTM: tergotrochanteral muscle
UTR: untranslated region
VLD: ventral lateral dendrite
