# Supplementary material for: The Nicotinic Acetylcholine Receptor Dα7 Is Required for an Escape Behavior inDrosophila
Source: PLoS Biol. 2006 Feb 28;4(3):e63. doi: 10.1371/journal.pbio.0040063 (PMC1382016; doi:10.1371/journal.pbio.0040063)

A

```
---------- --------ML VYGLGLLIMI PACAAGPHEK RLLHALLDNY
MSFPQPHSLP EATANGGRML VYGLGLLIMI PACAAGPHEK RLLHALLDNY
                            SP
NSLERPVVNE SDPLQLSFGL TLMQIIDVDE KNQLLITNIW LKLEWNDMNL
NSLERPVVNE SDPLQLSFGL TLMQIIDVDE KNQLLITNIW LKLEWNDMNL

RWNSSEFGGV RDLRIPPHRL WKPDVLMYNS ADEGFDGTYA TNVVVRNNGS
RWNSSEFGGV RDLRIPPHRL WKPDVLMYNS ADEGFDGTYA TNVVVRNNGS

CLYVPPGIFK STCKIDITWF PFDDQRCEMK FGSWTYDGFQ LDLQLQDEAG
CLYVPPGIFK STCKIDITWF PFDDQRCEMK FGSWTYDGFQ LDLQLQDEAG

GDISSFITNG EWDLLGVPGK RNEIYYNCCP EPYIDITFAI LIRRKTLYYF
GDISSFITNG EWDLLGVPGK RNEIYYNCCP EPYIDITFAI LIRRKTLYYF

FNLIVPCVLI ASMALLGFTL PPDSGEKLSL GVTILLSLTV FLNMVAETMP
FNLIVPCVLI ASMALLGFTL PPDSGEKLSL GVTILLSLTV FLNMVAETMP
          M1                           M2
ATSDAVPLLG TYFNCIMFMV ASSVVSTILV LNYHHRNPDT HEMSEWIRVI
ATSDAVPLLG KYFNCIMFMV ASSVVSTILV LNYHHRNPDT HEMSEWIRVI
                  M3
FLYWLPCILR MQRPGQVGYE CPPPPSSSSS SASGEKKQQI QNVELKERSS
FLYWLPCILR MQRPGQVGYE CPPPPSSSSS SASGEKKQQI QNVELKERSS

KSLLANVLDI DDDFRCNHRC ASATLPHQPT YYRTMYRQGD DGSVGPVGPA
KSLLANVLDI DDDFRCNHRC ASATLPHQPT YYRTMYRQGD DGSVGPVGPA

GPVVDGRLHE AISHTCLTSS AEYELALILK ELRWITEQLK KEDETSDITR
GPVVDGRLHE AISHTCLTSS AEYELALILK ELRWITEQLK KEDETSDITR

DWKFAAMVVD RLCLIIFTLF TIIATLAVLF SAPHFIFP-- --
DWKFAAMVVD RLCLIIFTLF TIIATLAVLF SAPHFIVSGV RG
                    M4
```

B

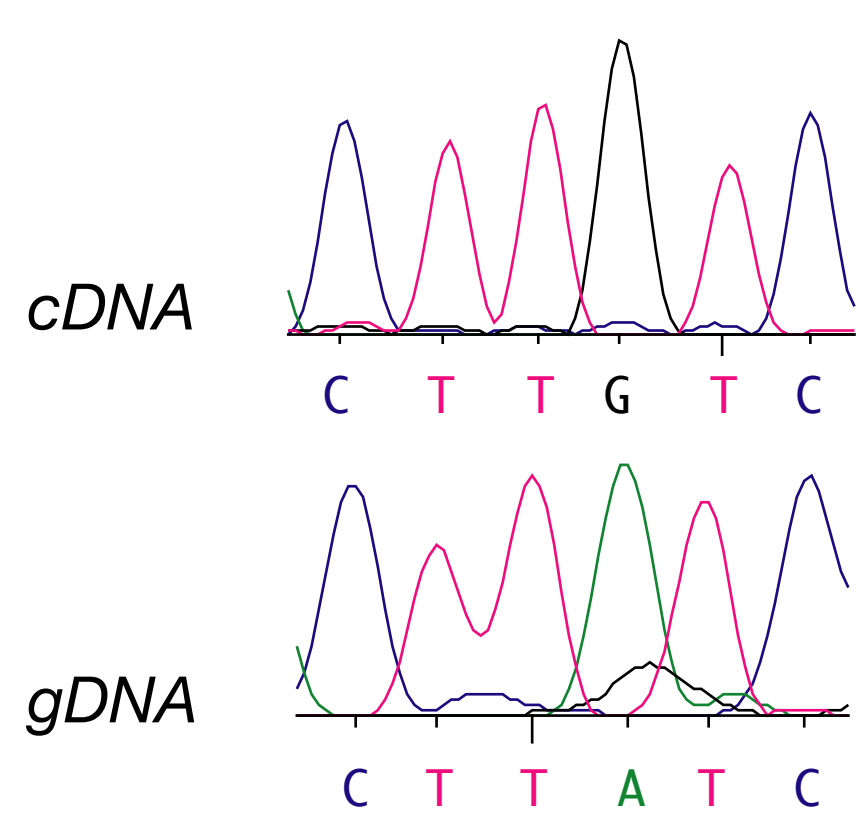

Supplement: Supplementary file 1 [file pbio.0040063.sg001.pdf]
