# Supplementary material for: The Nicotinic Acetylcholine Receptor Dα7 Is Required for an Escape Behavior inDrosophila
Source: PLoS Biol. 2006 Feb 28;4(3):e63. doi: 10.1371/journal.pbio.0040063 (PMC1382016; doi:10.1371/journal.pbio.0040063)
Supplement: Supplementary file 2 [file pbio.0040063.sd001.doc]

Protocol S1.

Expression of Dα7 in the Fly Central Nervous System

We localized the Dα7 protein in the adult central nervous system using immunohistochemistry. The specificity of the antibody can be judged by loss of staining in the CNS in the null mutant *PΔEY6* as described in the previous section. In these flies immunostaining is reduced to background levels throughout the nervous system with a single exception, the lamina (data not shown). This may be due to cross-reactivity of the antibody, possibly with another nAChR several of which show immunoreactivity in this region [1]. However, other evidence (see below) indicates that Dα7 may also be present in the lamina. Anti- Dα7 immunostaining shows that it is widely expressed in a variety of brain regions and that it is restricted to the neuropil. Below we summarize the basic expression pattern with respect to major brain areas:

## Deuterocerebrum and suboesophageal ganglion

In the deuterocerebrum (Supplemental Figure 2A) the glomeruli of the antennal lobes are labeled (al) as well as the anterior optic tubercle (aot). The suboesophageal ganglion is stained, possibly in the gustatory centers including cells that cross the midline (Supplemental Figure 2A) [2].

## Protocerebrum

The most prominently labeled structures in the protocerebrum is the dendrite of the giant fiber (Figure 7). Lateral to the giant fiber dendrites, the optic foci (of) that form the connections between the central brain and the optic lobes are labeled. In the dorsal protecerebrum the protocerebral bridge, a structure that is important for locomotion [3], is labeled as are the calyces (cx) of the mushroom bodies that receive cholinergic input from the antennal lobes via collateral projections of the antennoglomerular tract.

## Optic lobes

As shown in Supplemental Figure 2E, Dα7 staining is found in all neuropils of the optic lobes. In the lamina, anti- Dα7 staining is punctate, similar to the pattern of photoreceptor R1-R6 terminals on the lamina monopolar cells. A similar pattern is also seen in the second optic neuropil, the medulla, where Dα7 protein is organized in columns with staining in several distal layers and in layer M9 of the proximal medulla (me) [4]. A broad swathe of staining is found in the posterior half of the lobula while all four layers of the lobula plate are clearly labeled (lc).

## Ventral ganglion

Immunostaining in the ventral ganglion is restricted to specific regions of the neuropil. Prominent labeling is seen in the pro-, meso- and metathoracic segments as well as the abdominal ganglion including the ventrally located leg motor regions and the dorsal flight and neck motor areas (Supplemental Figure 2G).

While the antibody staining reveals the presence of Dα7 in many neuropils of the brain, it is often not possible to identify the cells expressing Dα7 based on synaptic staining. This is particularly important in the case of α7-like nAChRs because in the vertebrate CNS these receptors are often localized at the presynaptic terminal [5,6]. In order to overcome this limitation we generated a transgenic strain that expresses GAL4, a transcription activator from yeast that can be used to activate any gene cloned downstream of an Upstream Activating Sequence (UAS), under the control of enhancers that determine expression of the Dα7 receptor. Using P-element conversion [7], we replaced the SuPor-P P-element [8] KG3295 with a pGawB P-element [9] from a donor on the 3rd chromosome to create Dα7-GAL4. We tested Dα7-Gal4 by driving GFP fused to the transmembrane antigen mCD8 (UAS-mCD8-GFP) [10] and found high levels of expression in almost all of the areas labeled with the antibody (Supplemental Figure 2). However, since UAS-mCD8-GFP labels the entire cell while the antibody labels only Dα7 containing synaptic sites, GFP labeling was found in cell bodies and areas of the neuropil that were not labeled with the antibody. In the central brain the highest levels of expression are detected in the Kenyon cells, the intrinsic neurons of the mushroom bodies, antennal lobe neurons (Supplemental Figure 2 B,D) as well as the ellipsoid body and protocerebral bridge (pc) in the central complex. In the optic lobes, the lamina, medulla and lobula complex are labeled with prominent labeling of the distal and proximal layers including M9 (Supplemental Figure 2F) consistent with the pattern of immunostaining. In the ventral nerve cord we observe strong labeling in the flight neuropil and in cell bodies (dlm) of putative flight motorneurons (Supplemental Figure 2H).

**References**

1. Schuster R, Phannavong B, Schroder C, Gundelfinger ED (1993) Immunohistochemical localization of a ligand-binding and a structural subunit of nicotinic acetylcholine receptors in the central nervous system of *Drosophila melanogaster*. J Comp Neurol 335: 149-162.

2. Singh RN (1997) Neurobiology of the gustatory systems of *Drosophila* and some terrestrial insects. Microsc Res Tech 39: 547-563.

3. Strauss R (2002) The central complex and the genetic dissection of locomotor behaviour. Curr Opin Neurobiol 12: 633-638.

4. Fischbach KF, Dittrich APM (1989) The optic lobe of *Drosophila melanogaster.* I. A Golgi analysis of wild-type structure. Cell Tissue Res 258: 441-475.

5. McGehee DS, Heath MJ, Gelber S, Devay P, Role LW (1995) Nicotine enhancement of fast excitatory synaptic transmission in CNS by presynaptic receptors. Science 269: 1692-1696.

6. Gray R, Rajan AS, Radcliffe KA, Yakehiro M, Dani JA (1996) Hippocampal synaptic transmission enhanced by low concentrations of nicotine. Nature 383: 713-716.

7. Sepp KJ, Auld VJ (1999) Conversion of lacZ enhancer trap lines to GAL4 lines using targeted transposition in *Drosophila melanogaster*. Genetics 151: 1093-1101.

8. Roseman RR, Johnson EA, Rodesch CK, Bjerke M, Nagoshi RN, et al. (1995) A P element containing suppressor of hairy-wing binding regions has novel properties for mutagenesis in *Drosophila melanogaster*. Genetics 141: 1061-1074.

9. Brand AH, Perrimon N (1993) Targeted gene expression as a means of altering cell fates and generating dominant phenotypes. Development 118: 401-415.

10. Lee T, Luo L (1999) Mosaic analysis with a repressible cell marker for studies of gene function in neuronal morphogenesis. Neuron 22: 451-461.
